# Art, Healing, and Carceral Health

**DOI:** 10.3201/eid3013.AC3013

**Published:** 2024-04

**Authors:** Liesl Hagan, Andrew Durkin, Devon VanHouten-Maldonado, Byron Breedlove

**Affiliations:** Centers for Disease Control and Prevention, Atlanta, Georgia, USA; (L. Hagan, A. Durkin, B. Breedlove);; SkyART, Chicago, Illinois, USA (D. VanHouten-Maldonado)

**Keywords:** Carceral, youth, prison, jail, Art, Healing, and Carceral Health, SkyART, sexually transmitted infections, HIV, COVID-19, respiratory infections, bacteria, viruses, about the cover

**Figure:**
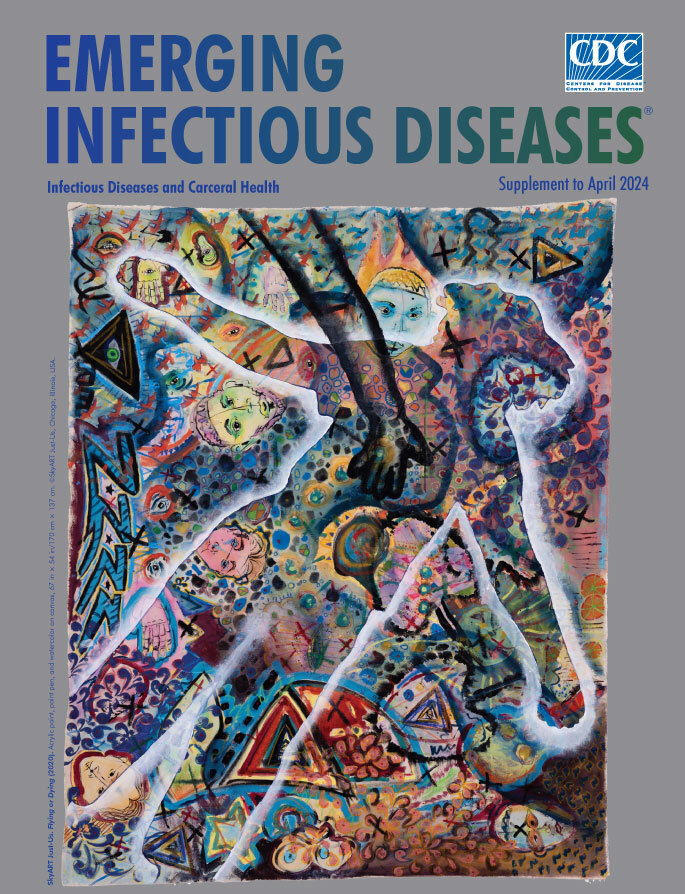
**SkyART Just-Us. *Flying or Dying* (2020).** Acrylic paint, paint pen, and watercolor on canvas, 67 in x 54 in/170 cm x 137 cm. © SkyART Just-Us, Chicago, Illinois, USA.

People who are incarcerated are among the most marginalized groups in our society, and their lives—and their experiences with infectious diseases—are often unseen behind the walls of prisons and jails. *Flying or Dying*, the cover art for this supplement issue of *Emerging Infectious Diseases*, was created as part of a 2022 exhibit named *Can You See Me*? and organized by SkyART, a nonprofit organization that provides art programs and creative arts therapy for young artists in Chicago’s South and West Side communities. Since 2018, SkyART has worked in youth detention centers in the Chicago area, including several facilities operated by the Illinois Department of Juvenile Justice.

*Flying or Dying* is a collaborative mural that was displayed in the Weinberg/Newton Gallery, Arts + Public Life, and SkyART studios in Chicago. The *Can You See Me?* exhibit featured works by more than 50 artists and organizations, including youth held in Chicago-area detention facilities. The objective of *Can You See Me?* was to highlight the humanity and potential of incarcerated youth and to demonstrate how art can be a tool for healing.

*Flying or Dying*, created by eight young artists over the course of several months, features many layers, evolving from graffiti to portraits, that tell many stories, both hopeful and harrowing. The white outline of a body that overlays most of the piece challenges the viewer to determine whether the figure is a chalk outline—another faceless and nameless victim of violence—or a person with their arms spread wide as they take flight and soar over the violence traumatizing their community. Are they fallen, or are they flying? Because state law protects the identity of minors who are detained or charged with crimes, the artists are not named. However, the outlines of their bodies on the canvas bring them physically into the exhibition space, reminding viewers that they are real people. The patterns, shapes, texts, and images create a more nuanced and complete portrait of these young men as full and complex individuals, more than the crimes for which they were confined.

SkyART’s Just-Us programs are led by art therapists, social workers, and licensed counselors who use creative techniques to help young persons express, process, and heal from trauma they have experienced. Incarcerated youth are affected by an average of six adverse childhood experiences, often including gun violence, abuse, and neglect, which negatively affect their health outcomes. When young persons enter the criminal justice system, they are often required to participate in traditional cognitive behavioral therapy or talk therapy, which can be difficult for some youth. Alternative creative modalities such as art therapy offer young persons a different way to process emotions that can be difficult to put into words.

According to the Prison Policy Initiative, more than 48,000 youth are confined in the United States on any given day. The youth incarceration rate in the United States is higher than in any region of the world as defined by the United Nations and is 11 times higher than in western Europe or Asia. Data collected in 2019 by the US Department of Justice show that young persons of color are disproportionately represented; detention rates among Black youth are six times higher and among Latinx youth two times higher than among white youth. Confinement during adolescence has been associated with poorer mental and physical health (including higher prevalence of infectious diseases) during adulthood and a shorter life expectancy.

CDC data from 2019 show that rates of sexually transmitted infections, including chlamydia and gonorrhea, are higher among youth entering confinement facilities than among youth in the community. Youth in detention facilities also commonly report a history of sexual behavior associated with increased risk of acquiring HIV, including having had unprotected sex and multiple partners. However, HIV prevalence in this population is unclear because of variable screening practices at the time of entry, a common public health challenge associated with carceral health overall. Youth held in detention facilities are also at increased risk for respiratory diseases, including COVID-19, because of the congregate living environment. In addition to infectious diseases, mental health among youth who are confined is a serious concern. The 2016 US Bureau of Labor Statistics National Longitudinal Survey of Youth found that young persons confined for one year or longer were four times more likely to experience depression and two times more likely to have suicidal thoughts during adulthood compared with their peers who were not confined.

Similar to the objective of SkyART’s *Can You See Me?* exhibit, this supplement in *Emerging Infectious Diseases* portrays the lives of persons who are incarcerated and brings them into greater prominence. Each article in the supplement illustrates ways in which the experiences of incarcerated persons, as well as staff working in carceral facilities, are critical components of infectious disease prevention and public health broadly. Within public health practice, the same question resonates: Can we “see” persons confined in carceral facilities, and do we see them as part of our work? Just as important, can persons living and working in these facilities see themselves in our public health and infectious disease prevention strategies? To build a future where people can be safe and access high-quality healthcare during confinement, and where they can live healthy lives afterward, the answer to “Can you see me?” must be “Yes.”
